# Enhancing coronary artery plaque analysis via artificial intelligence-driven cardiovascular computed tomography

**DOI:** 10.1177/17539447241303399

**Published:** 2024-12-03

**Authors:** Jeffrey Xia, Kinan Bachour, Abdul-Rahman M. Suleiman, Jacob S. Roberts, Sammy Sayed, Geoffrey W. Cho

**Affiliations:** David Geffen School of Medicine at UCLA, Los Angeles, CA, USA; David Geffen School of Medicine at UCLA, Los Angeles, CA, USA; David Geffen School of Medicine at UCLA, Los Angeles, CA, USA; David Geffen School of Medicine at UCLA, Los Angeles, CA, USA; David Geffen School of Medicine at UCLA, Los Angeles, CA, USA; David Geffen School of Medicine at UCLA, 100 Medical Plaza, Suite 545, Los Angeles, CA 90024, USA; Cardiovascular Research Foundation of Southern California, Beverly Hills, CA, USA

**Keywords:** APC, artificial intelligence, CAC, CAD, CCTA, CONFIRM, DL, FFT-CT, ML

## Abstract

Coronary computed tomography angiography (CCTA) is a noninvasive imaging modality of cardiac structures and vasculature considered comparable to invasive coronary angiography for the evaluation of coronary artery disease (CAD) in several major cardiovascular guidelines. Conventional image acquisition, processing, and analysis of CCTA imaging have progressed significantly in the past decade through advances in technology, computation, and engineering. However, the advent of artificial intelligence (AI)-driven analysis of CCTA further drives past the limitations of conventional CCTA, allowing for greater achievements in speed, consistency, accuracy, and safety. AI-driven CCTA (AI-CCTA) has achieved a significant reduction in radiation exposure for patients, allowing for high-quality scans with sub-millisievert radiation doses. AI-CCTA has demonstrated comparable accuracy and consistency in manual coronary artery calcium scoring against expert human readers. An advantage over invasive coronary angiography, which provides luminal information only, CCTA allows for plaque characterization, providing detailed information on the quality of plaque and offering further prognosticative value for the management of CAD. Combined with AI, many recent studies demonstrate the efficacy, accuracy, efficiency, and precision of AI-driven analysis of CCTA imaging for the evaluation of CAD, including assessing degree stenosis, adverse plaque characteristics, and CT fractional flow reserve. The limitations of AI-CCTA include its early phase in investigation, the need for further improvements in AI modeling, possible medicolegal implications, and the need for further large-scale validation studies. Despite these limitations, AI-CCTA represents an important opportunity for improving cardiovascular care in an increasingly advanced and data-driven world of modern medicine.

## Introduction

There have been significant recent advancements in artificial intelligence (AI)-driven cardiovascular imaging. AI techniques such as machine learning (ML) and deep learning (DL) neural networks are increasingly being studied to interpret the immense data acquired through various cardiovascular imaging modalities including coronary computed tomography angiography (CCTA).

Despite advances in diagnostics and therapeutics in cardiovascular medicine, heart disease remains the leading cause of death in the United States, accounting for nearly one million deaths in 2020, with coronary heart disease as the leading cause.^1^ CCTA is a noninvasive CT imaging modality of cardiac structures and vasculature that is now considered equivalent to invasive coronary angiography testing for evaluation of coronary artery disease (CAD) in several guidelines. The rise of AI in CCTA (AI-CCTA) demonstrates promising results in several avenues, such as improvements in the efficiency of image acquisition, post-processing time, diagnostic ability, consistency, and even insight into risk stratification and prognostication of outcomes.^2–4^

This review provides an overview of recent updates in the applications of AI-CCTA with a focus on CAD including fundamentals in AI, CCTA image acquisition, coronary artery calcium (CAC), coronary stenosis, adverse plaque characteristics (APCs), and fractional flow reserve CT (FFR-CT).

## Utilization of CCTA in modern medicine

As the quality of evidence and technology for CCTA advances, there has been increasing inclusion and expansion of CCTA utilization in international guidelines for CAD. In 2016, CCTA emerged as a first-line diagnostic modality for CAD in the UK’s National Institute for Health and Care Excellence (NICE) guidelines. Several studies investigating the utilization of CCTA such as the 5-year outcomes from SCOT-HEART, CREDENCE, CONSERVE, and ISCHEMIA demonstrated the versatility and utility of CCTA for CAD.^5–8^ The data from these studies stimulated clinical change in CAD management with associated reduction in mortality, and with superiority to functional stress testing for significant coronary stenoses serving as a gatekeeper for diagnostic invasive coronary catheterization.^5–8^ In 2019, the European Society of Cardiology updated its guidelines for chronic coronary syndrome management, upgrading the recommendations for CCTA as equivalent to functional stress testing as first-line diagnostic testing for CAD (class I recommendation) and CCTA as an alternative to invasive coronary angiography in the setting of non-diagnostic functional testing if adequate imaging quality is achievable (class I recommendation).^9^ In 2021, the American College of Cardiology/American Heart Association updated guidelines for the evaluation and diagnosis of chest pain with a class I recommendation for CCTA to exclude plaque and obstructive CAD in intermediate-risk patients.^10^ With further alignments between international guidelines, the utilization of CCTA is expected to rise, necessitating technological advances to keep up with demand while ensuring continued safety for patients obtaining these studies.

As a noninvasive imaging modality capable of both anatomical characterization of plaque (quantitative and qualitative) and functional testing (FFR-CT), CCTA now makes it simpler and safer for temporal tracking of plaque while optimizing medical therapy through long-term serial CT exams compared to intravascular ultrasound for plaque analysis. Examples of the application of serial CCTA include the PARADIGM and EVAPORATE studies.^11,12^ PARADIGM demonstrated the phenotypic changes of coronary plaque associated with statins including increased plaque calcification and reduction of high-risk plaque features over an interscan interval ⩾2 years.^11^ EVAPORATE demonstrated significant regression of low-attenuation plaque (LAP) volume due to icosapent ethyl with an interscan interval of 9 and 18 months.^12^ Furthermore the noninvasive nature of CCTA may allow for reduced procedural risks as in the DISCHARGE trial that demonstrated a reduced rate of procedure-related complications in patients receiving CCTA prior to undergoing invasive coronary angiography.^13^

## AI sub-types in cardiac CT

AI is the generalized term describing computational processes that aim to imitate human intelligence, such as learning, reasoning, and decision-making.^14^ ML is a subset of AI and utilizes sophisticated statistical analysis of an input data set to produce output predictions or decisions.^14^

There are four types of ML algorithms: supervised learning, unsupervised learning, semi-supervised, and reinforcement learning.^14^ Supervised learning involves the use of human-labeled data to evaluate outcomes which the ML algorithm learns from. Unsupervised learning is a more independent ML sub-type in which ML occurs without the use of labeled data. Semi-supervised learning occurs when a model uses a hybrid approach of both methods. Reinforced learning utilizes rewards and penalties within the algorithm to maximize cumulative rewards.^14,15^

DL is a sub-type of ML and uses all types of learning in a hierarchical structure called neural networks allowing for improved analysis of various features of an image presented to the model. Convolutional neural networks (CNNs) utilize layers of neural nodes when processing an input image to allow for enhanced pattern recognition. DL paired with CNN allows for potentially advanced interpretation of images than conventional supervised learning models.^16,17^ The limitations of AI include the necessity for high-quality datasets to train AI models (time intensive to accrue), difficulty with novel or rare imaging findings, interpretation of multidimensional or nonlinear images, and the ability to externally validate results from one dataset to larger populations.^15^

Currently in cardiovascular imaging, supervised learning is the most common method for the interpretation of images, allowing for human labeling of specific imaging features such as plaque characteristics and degree of stenosis which trains the ML model.^18^ Once trained with the learning dataset, the model attempts to interpret new CCTA images. The application of AI-CCTA is rapidly expanding due to the inherently graphical nature and data-driven aspects of CT imaging.

## AI in technical image acquisition and quality in CCTA

One of the greatest theoretical risks of CCTA is ionizing radiation exposure which may predispose to cancer. PROTECTION VI was an international radiation dose survey on CCTA which revealed a 78% reduction in radiation dosage when compared to a prior survey in 2007.^19^ During this 10-year interval, the primary strategies to reduce radiation were non-AI, comprising hardware (CT gantry modifications, tube voltage adjustments), software (iterative image reconstruction), and procedural (ECG-gating) optimization techniques.^19^ Further reduction in radiation dose and image quality is limited by increasing image noise and processing time. Studies utilizing AI-driven denoising techniques have been shown to decrease the amount of radiation needed for CCTA without sacrificing image quality (Table 1). Almost all studies in Table 1 achieved sub-millisievert and dose length product (DLP) <100 mGy*cm radiation doses utilizing AI processing of CCTA data with no significant difference, or even improved, image quality compared to higher radiation doses. In comparison, the median DLP in 2017 from PROTECTION VI was 195 mGy*cm (IQR: 110–338 mGy*cm) which is equivalent to 2.7 or 5.1 mSv using effective dose conversion factors of 0.014 or 0.026 mSv/mGy*cm, respectively.^19^ In addition, some studies achieved lower doses of contrast administered with AI-CCTA data processing without compromising image quality.^20,21^ Limitations to these studies include variability of CT scanners, variability in study protocols, lack of randomization, small sample sizes, and variability in human adipose distribution.

**Table 1.** Studies investigating the radiation dosage reduction efficiency utilizing artificial intelligence in the processing of coronary CT angiogram data.

Study	Year	N	Focus	Tube voltage (kV)	Networks	Algorithm	Comparison group	Effective dose (mSv)	Effective dose (DLP, mGy*cm)	Degree of radiation dose reduction (%)
Liu et al.^22^	2020	70	Image quality	80, 100	DCNN	DL-GAN	IR	N/A	66	55
Bernard et al.^23^	2021	296	Image quality	120	DCNN	AiCE	AIDR-3D	1.5	106.4	40
Benz et al.^24^	2022	50	Image quality	120	DCNN	DLIR-H	ASiR-V	0.8	31	43
Li et al.^20^	2022	100	Image quality	70, 120	DCNN	DLIR-H	ASiR-V	0.75	53.44	54.5
Wang et al.^25^	2022	60 (all BMI ⩾ 30)	Image quality	100, 120	DCNN	DLIR-M DLIR-H	ASiR-V	1.8	69.4	40
Wang et al.^26^	2022	80	Image quality	70 (BMI < 26), 80 (BMI⩾ 26)	DCNN	DLIR-M DLIR-H	ASiR-V FBP	0.93 (70) 2.35 (80)	63.17 (70) 168 (80)	N/A
Demircioglu et al.^27^	2023	298	Delimitation	90, 120	CNN	Cascade R-CNN VFNet YOLOX	Radiologists	7.3	N/A	12.6
Li et al.^21^	2023	100 (all BMI > 26)	Image quality	80, 120	DCNN	DLIR-H	ASiR-V	1.01	71.42	45

AiCE, advanced intellegent clear-IQ engine; AIDR, advanced interative dose reduction; ASiR, adaptive statistical interative reconstruction; BMI, body mass index; CNN, convolutional neural network; DCNN, deep convolutional neural network; DLIR, deep learning iterative reconstruction; DLP, dose length product; IR, iterative reconstruction.

Observed from survey data from PROTECTION VI, the use of iterative reconstruction (IR) in CCTA imaging surpassed the conventional filtered back projection (FBP) technique in 2017, with 83% of surveyed centers utilizing IR. IR incorporates an FBP estimate and compares CCTA data in an iterative process while applying denoising models to improve image quality. This iterative process is attractive within the AI space as it is data driven with parameters easily adjustable using DL models, meshing the iterative process with multiple processing layers of neural networks. The current status of DL iterative reconstruction (DLIR) is early but rapidly evolving. In a study that formally assessed DLIR in comparison to conventional statistical reconstruction (ASiR) with invasive coronary angiography as a standard reference, DLIR achieved 43% noise reduction while increasing image quality by 138%; and there were no significant differences in accuracy (82% vs 80%), AUC (0.826 vs 0.802), sensitivity (92% vs 88%), and specificity (72% vs 73%) with high inter-reader and intra-reader reliability (ICC 0.90 and 0.86, respectively).^28^ There is great potential for DLIR in a number of applications, as demonstrated in Table 1 by the studies utilizing DLIR to reduce radiation dose per scan. Another application is the utilization of DLIR to evaluate in-stent restenosis by correcting for stent-related blooming artifacts. In one study, CCTA with DLIR was used to assess in-stent restenosis with invasive coronary angiography as the reference standard; sensitivity was moderate (50%–62.5%) with high specificity (90.1%–95.5%) and comparable positive predictive value (PPV) (71.4%–80%) and negative predictive value (NPV) (84%–87%).^29^

Imaging a dynamically moving organ such as the heart may result in the occurrence of motion artifact, especially when the heart rate is not low enough or the CT scanner has a less efficient temporal resolution. ML provides the opportunity to correct for motion artifacts in post-processing. One ML method for motion artifact reduction is utilizing generative adversarial networks (GAN; Table 2). With GAN in the context of CCTA motion reduction, a discriminator neural network attempts to decipher whether an image is a real motion-free image supplied by a domain versus a generated artificial image without motion artifact. These inputs reiterate and train opposing networks for which the parameters can be used for post-processing motion artifact reduction. In one study involving paired motion-affected and motion-free CCTA images, GAN-driven motion artifact reduction significantly improved quantitative quality analysis measures including peak signal-to-noise ratio, structural similarity (SSIM), dice similarity coefficient, and Hausdorff distance with significantly improved rater subjective assessment for motion improvement.^30^ Furthermore, this study compared GAN-generated CCTA images and motion-affected CCTA images to invasive coronary angiographic reference standards, finding significant improvement in accuracy for diagnosing no coronary stenosis (40%–81%), <50% stenosis (47%–85%), and ⩾50% stenosis (62%–70%).^30^ The limitations of current studies regarding AI motion artifact reduction include the retrospective nature of studies and small sample sizes.

**Table 2.** Studies investigating motion artifact reduction efficacy of artificial intelligence processing of coronary CT angiogram data.

Study	Year	N	Network	Generator	Discriminator	Peak signal-to-noise ratio [IQR]	Structural similarity (SSIM) [IQR]	Dice similarity coefficient [IQR]	Hausdorff distance [IQR]
Zhang et al.^30^	2022	313	CNN, GAN	U-Net	PatchGAN	26.1 [24.4–27.5]	0.860 [0.830–0.882]	0.783 [0.714–0.825]	4.47 [3.00–7.07]
Ren et al.^31^	2022	97	CNN, GAN	U-Net	PatchGAN	N/A	0.87 ± 0.06	0.84 ± 0.08	N/A
Deng et al.^32^	2022	60	CNN, GAN	U-Net	CNN	24.96 ± 1.54	0.769 ± 0.055	N/A	N/A

CNN, convolutional neural network; GAN, generative adversarial network.

Another area of interest in AI-CCTA image processing is segmentation. Image segmentation is the process of separating an image into regions of interest (ROI) to allow for more efficient and meaningful image analysis (Table 3).^33^ In AI, neural networks can be programmed to analyze CCTA images and analyze minute details of coronary pathologic anatomy including localization of atherosclerotic plaques/stenoses, morphologic features of plaque, and degree of plaque calcification. In CLARIFY, the performance of CNN-analyzed coronary atherosclerosis on CCTA images was compared to level 3 expert CCTA readers, finding 78% overall agreement in CAD-RADS with the greatest discrepancy at CAD-RADS 0 and AI CAD-RADS 1.^34^ In lesions with potential interventional treatment (>70% stenosis), there was >99% consensus between AI and level 3 expert readers, with a per-patient sensitivity of 88.9%, specificity 99.6%, PPV 88.9%, and NPV 99.6%.^34^ Furthermore, the AI system processed CCTA images in 9.7 ± 3.2 min.^34^ Improvement of efficiency by AI-assisted CCTA analysis has been estimated to be 69%–80% from human analysis.^35,36^ These outcomes of high diagnostic performance and efficiency were also seen in a post hoc analysis of CREDENCE.^37^ Limitations of these studies include lack of standard reference with invasive coronary angiography, and unknown prognostic implications of CCTA at the time of study.

**Table 3.** Studies investigating the data processing efficiency of artificial intelligence segmentation analysis of coronary CT angiogram data.

Study	Year	*N*	Processing time (min)
Choi et al.^34^	2021	232	9.7 ± 3.2
Griffin et al.^37^	2023	303	10.3 ± 2.7
Liu et al.^36^	2021	165	2.3 ± 0.6
Jonas et al.^38^	2022	303	10.3 ± 2.7
Meng et al.^39^	2023	256	4.97 [IQR: 4.60–5.42]

## AI in CAC scoring and calcified plaque analysis

The quantification of CAC, a key component of atherosclerosis, is thought to predict the severity of CAD.^40–43^ Historically, chest radiography and fluoroscopy were the most common modalities used to detect CAC.^41^ However, it was not until the advent of CT that allowed for the feasibility of CAC quantification.^41^ Several major studies, such as multi-ethnic studies of atherosclerosis, Heinz Nixdorf Recall (HNR), and Framingham Heart have shown that high CAC scores are associated with the presence of CAD and independently predict cardiovascular events.^44–46^ The most common method of scoring CAC is known as the Agatston score, which is a semi-automated tool that grades CAC using calcium density and area. This method can be time-consuming, likely preventing widespread clinical application.^41,43,47^

AI algorithms are increasingly being studied to improve the efficiency of CAC scoring. Historically, CAC scores were obtained using ECG-gated cardiac CT. However, an emerging concept is AI-CAC scoring utilizing non-gated, non-contrasted general CT chest imaging. A study investigating DL-assisted CAC scoring on both non-gated and ECG-gated non-contrasted chest CT revealed a strong correlation coefficient of 0.964 suggesting DL-assisted CAC scoring of non-gated CT chest imaging may be comparable to ECG-gated studies.^48^ Another study compared an AI-CAC scoring model against the gold standard of blinded radiologists with 5 years of experience and demonstrated that the AI model both reliably and accurately calculated CAC scores and classified patients into the appropriate risk stratification groups.^49^ In addition, even compared to expert human level 3 CT readers, it was shown that the AI platform performed equally well, and even additionally captured some calcium missed by human analysis.^50^ The application of AI-CAC extends into CNNs which one study demonstrated a correlation coefficient of 0.93 between CNN-assisted Agatston calculation compared to known human-calculated CAC scores without the need for prior data segmentation of CAC.^51^

AI may also be useful in calculating CAC from CCTA studies. One study compared CNN-assisted CAC scoring of CCTA against paired manual CAC scoring of dedicated coronary calcium scans which revealed a strong correlation between these modalities with comparable risk stratification outputs.^52^ Another study compared DL-assisted CAC scoring against manual CAC scoring of CCTA imaging data only and demonstrated that the DL algorithm was highly sensitive, specific, and with strong diagnostic accuracy >90%.^53^ These findings suggest that AI-CAC scoring may be useful in optimizing efficiency in CAC scoring.

Aside from CAC scoring, AI-assisted analysis of calcified plaque is an area of ongoing research. A recent study performed in patients with pre-existing end-stage renal disease and a mean CAC greater than 2000 found that plaque calcification did not significantly affect the specificity of CCTA. In addition, over 94% of samples were deemed analyzable using automated AI-enabled software despite a significant CAC burden.^54^ Another study tested a 3D CNN used for plaque segmentation and classification on a pre-existing dataset of CCTA volumes. AI-assisted analysis of calcified plaque was found to have a dice score of 0.83 suggesting strong overlap between AI-assisted analysis and manual analysis.^55^ Aside from direct AI assistance in the analysis of calcified plaque, AI models may indirectly assist through calcium de-blooming via GANs. A recent study utilized GAN-processed images for calcium de-blooming of 50 patients with calcified plaques found a significant reduction in false-positive rates compared to the original, unprocessed images. The study also found a significant improvement in the specificity and PPV of CCTA.^56^

## AI and coronary stenosis in CCTA

Historically, CAD was assessed through invasive coronary angiography. However, the rapid development of CCTA allows for increasingly advanced visualization of coronary anatomy, the identification of luminal stenosis, plaque burden, and high-risk plaque features.^57,58^

**Figure 1.**
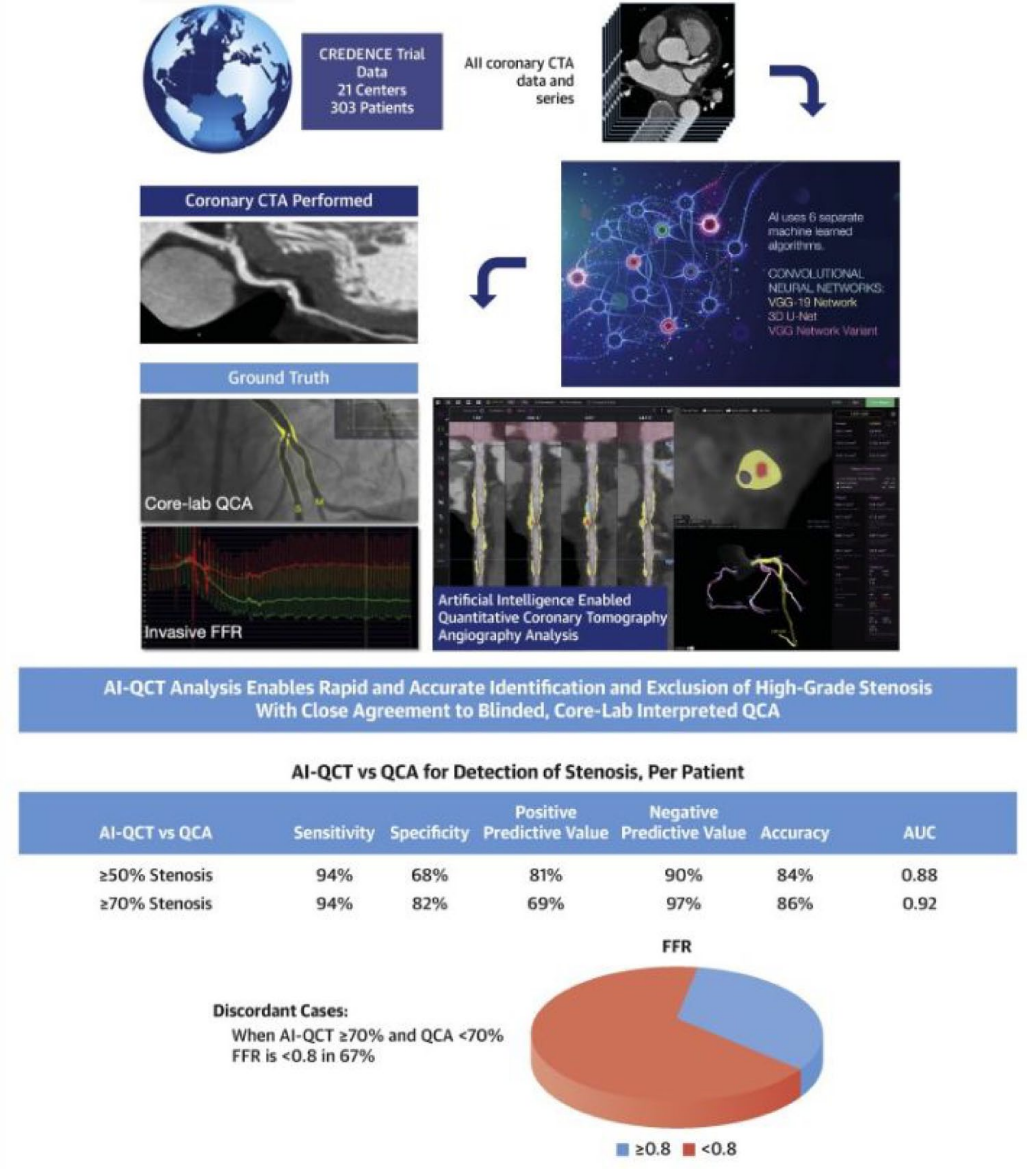
Study design flow schematic of an artificial intelligence CCTA investigation of coronary artery stenosis. Source: Reused with permission from Griffin…Earls et al.^37^ CCTA, coronary computed tomography angiography.

The findings of the CLARIFY trial are particularly important given the large variability in the manual quantification and categorization of severe stenosis. This is particularly evidenced in the PROMISE trial which compared local site interpretations with blinded, off-site interpreters with variable CCTA reading experience which demonstrated significant variability in the interpretations of CCTA particularly in the off-site interpreters blinded to clinical data.^59^ PROMISE highlighted the difficulty of standardized and accurate human interpretations of CCTA particularly at high-volume centers where incorporation of clinical data may not always be feasible for the interpretation of the study.^59^ Aside from CLARIFY, several other studies have validated the use of AI in the interpretation of CCTA.^39,60^ One study found that AI-assisted analysis using DL was non-inferior to manual interpretation as it relates to stent segmentation >90% sensitivity, specificity, PPV, and NPV for the diagnosis of ⩾50% stenosis in the internal dataset with comparable performance in external validation testing.^39^ Another study compared ML techniques to expert CCTA interpretation for the detection of coronary stenosis ⩾25% and demonstrated the ML techniques had high sensitivity (93%) and specificity (95%; Figure 1).^60^

## AI-driven quantification and prognostication of APC

Invasive and pathologic studies have determined high-risk anatomic plaque features central to the processes of acute coronary syndromes (ACS) and sudden cardiac death.^61^ Among high-risk plaque lesions, common characteristics include plaque burden, thin-cap fibroatheroma, positive arterial remodeling, necrotic cores, and spotty calcifications.^62^ Invasive data demonstrated that the majority of plaques implicated in ACS are non-obstructive in terms of stenosis severity, with high-grade stenoses comprising less than one-third of culprit lesions.^63^ Therefore, characteristics beyond the severity of stenosis are needed to identify high-risk plaques, which is achievable with CCTA.

Quantitative evaluation of APCs on CCTA can identify patients at risk for cardiac events resulting from high-risk plaque ruptures. In addition to measuring luminal diameter narrowing to detect coronary stenoses, advances in CCTA have allowed for the development of APC analysis including aggregate plaque volume, positive arterial remodeling, LAP as markers for necrotic lipid-laden intra-plaque core, and spotty intra-plaque calcification. One study identified low-attenuation plaque burden as the strongest predictive APC for fatal or non-fatal MI.^64^ Studies have demonstrated that even in non-obstructive lesions, APCs correlated with adverse outcomes.^65–67^

Studies have demonstrated that APCs by CT improve early identification of coronary lesions that cause ischemia supporting a potential preventative role.^68^ Studies have further demonstrated that CT perfusion imaging and APCs identified on CCTA can detect ischemic stenosis when using single-photon-emission CT as a reference for these lesions.^69^

**Figure 2.**
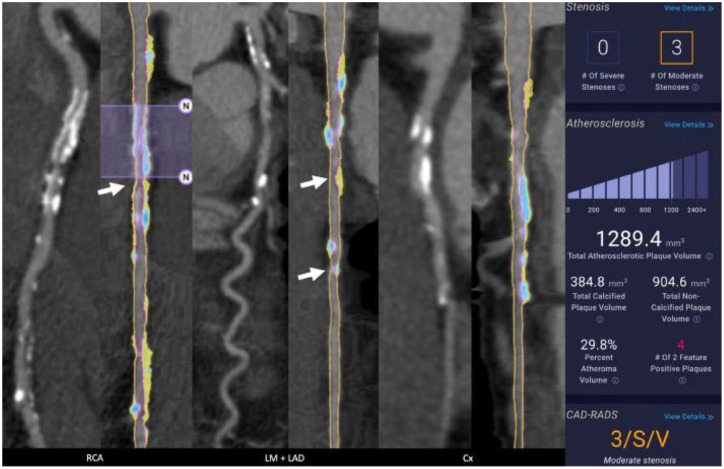
Example of adverse plaque characteristic analysis in a patient with end-stage renal disease. Three groups of two images of the right coronary artery (RCA), left main/left anterior descending (LM/LAD), and circumflex are presented. Each group’s left image is a curved MPR of the CCTA, and the right images are straightened MPR with color plaque overlay (red = LDNCP, yellow = NCP, blue = CP). A 20 × 3.0 mm^2^ stent is present in the proximal RCA and was excluded from QCT analysis (purple overlay with “N” markers, second image from left). This is the only exclusion in this patient, and it represented 2.82% of the coronary vessels measuring 2 mm or greater. Note that a 51% stenosis was depicted distal to the stent (arrow). Two moderate stenoses of 61% and 55% were depicted in the mid LAD (fourth image, arrows). Non-obstructive disease was present in the circumflex. The patient’s plaque volume was high at 1289.4 mm^3^, CP 384.8 mm^3^, NCP 904.6, total PAV was 29.8%, and four two-feature HRPs were depicted. Source: Reused with permission from Cho et al.^54^ AUC, area under curve; CCTA, coronary computed tomography angiography; CP, calefied plaque; HRP, high risk plaque; MI, myocardial infarction; MPR, multiplanar reformat; LDNCP, low-density non-calcified plaque; NCP, non-calcified plaque; PAV, plaque atheroma volume; QCT, quantitative computive tomography.

While APCs have proven clinically useful in predicting high-risk plaque lesions, the parameters measured to assess these characteristics cannot be readily determined due to time-consuming calculations. As a result, studies have investigated the utility of AI-CCTA to evaluate plaque characteristics. Several programs have been developed to further develop AI-CCTA. Among programs with FDA approval are Cleerly and HeartFlow which serve as AI-based digital platforms to improve clinician identification of high-risk plaques. Studies by Cho et al.^70^ using these platforms demonstrate that it is possible to perform serial analysis of APC changes over time to provide insight into the prognostication of cardiovascular events. Furthermore, it was also shown that plaques that would otherwise be difficult to quantify by human reading due to high calcium and plaque burden can be characterized accurately via AI interpretation.^54^ Other approaches to AI-CCTA utilize a radiomics approach to analyze APC. Through radiomics, quantitative data are analyzed by extracting numerous features from images by focusing on the ROI and quantifying textural information by extracting spatial distribution of Hounsfield or signal intensities and relationships.^71–73^ This radiomics approach has been shown to increase the diagnostic accuracy of CCTA in the identification of high-risk plaque characteristics.^73–75^ The segment involvement score (SIS) has also been developed as a semiquantitative measure of the extent of atherosclerosis burden by CCTA. Studies have demonstrated that the extent of CAD as quantified by SIS on CCTA provides strong predictors of cardiovascular events (Figure 2).^76^

## AI and functional flow reserve in CCTA

FFR-CT has increased importance in assessing ischemic lesions in arteriosclerotic plaques. CCTA is extremely useful in assessing vessel anatomy, which helps exclude significant CAD lesions (>50% narrowing) in low- and intermediate-risk populations.^77^ However, it has poor specificity in assessing obstructive CAD owing to calcium blooming (overestimation of calcium plaque) or inability to assess stenosis impact on flow, which is a more reliable predictor of ischemia.^78^ FFR-CT quantification addresses this limitation without the use of additional image acquisition or any stress agents. For example, in the landmark ADVANCE trial, the authors demonstrated the addition of FFR-CT to CCTA-modified clinical decisions in up to two-thirds of patients predicting the need for revascularization.^79^ When compared to the standard of care in intermediate stenotic lesions, the TARGET trial showed on-site CT-FFR reduced the number of patients who underwent invasive angiography with non-obstructive CAD.^80^

FFR assesses the ratio of measured maximal hyperemic blood flow distal to a lesion in an artery, usually assessed invasively by infusion of adenosine, to a theoretical maximal hyperemic flow in the same artery.^81^ FFR-CT utilizes the patient-specific three-dimensional anatomic model of coronary vessels and mathematical data and algorithms to approximate CT data flow, resistance, and pressure during rest and hyperemia to calculate an FFR. The limitations of the conventional FFR-CT model include precision of three-dimensional models, and available computational power/time. Another model has been introduced that utilized hybrid reduced-order (mainly for healthy and non-stenotic regions) and full-order models to allow for fast flow computation in less than 1 h (Table 4).^82,83^

**Table 4.** Key trials investigating CT-FFR.

Trial	Objective	Study design	Key findings
ADVANCE^79^	Evaluate the diagnostic value of FFR-CT in noninvasive assessment of CAD	Multicenter, prospective international registry (5000+ patients)	FFR-CT + CCTA improves decision-making and outcomes, reducing unnecessary angiography and optimizing revascularization
TARGET^80^	Comparing CCTA/CT-FFR strategy (group A) versus usual care (group B) on intermediate- to high-risk patients with suspected CAD who undergo clinically indicated diagnostic evaluation	Multicenter, prospective, open-label, and randomized controlled trial	CT-FFR reduced patients undergoing invasive coronary angiography without obstructive disease or requiring intervention within 90 days but increased revascularization overall without improving symptoms or quality of life or reducing major adverse cardiovascular events
DISCOVER-FLOW^84^	Compare diagnostic performance of FFR-CT versus goal standard invasive FFR	Prospective study, 103 patients	FFR-CT had high sensitivity and specificity in identifying ischemia-causing stenosis when compared to invasive FFR
NXT^85^	Compare diagnostic performance of FFR-CT versus goal standard invasive FFR	Prospective study, multicenter (254 patients)	FFR-CT showed a high correlation with invasive FFR in identifying hemodynamically significant stenoses

CAD, coronary artery disease; CCTA, coronary computed tomography angiography; FFR-CT, fractional flow reserve CT.

Successful utilization of AI further improves computational efficiency without significantly sacrificing accuracy. One study used a multilayer neural network with supervised learning of input of coronary vascular anatomy paired with output FFR-CT values and demonstrated reduced computation time by over 80-fold, allowing for physician-driven, near-real-time assessment of FFR-CT, all via standard workstations; The AI-FFR-CT achieved 81.6% sensitivity, 83.9% specificity, and 83.2% accuracy.^86^

A meta-analysis that included FFR-CT data from DISCOVER-FLOW, DeFACTO, and NXT multicenter trials demonstrated a combined sensitivity of 0.90 (95% CI: 0.85–0.93) and specificity of 0.82 (95% CI: 0.68–0.76) for ischemia detection, however, did not include AI-FFR-CT.^87^ MACHINE compared the ML-based approach against computational flow dynamics (CFD)-based FFR-CT with invasive FFR as the reference which demonstrated both ML-FFR-CT and CFD-FFR-CT outperformed visual CCTA assessment (AUC: 0.84 compared to 0.69, *p* < 0.001) with ML-FFR-CT improving accuracy from 58% to 78%.^88,89^

Furthermore, CCTA accuracy declines in the setting of extensive plaque. However, with ML-FFR-CT, the accuracy, sensitivity, and specificity of FFR-CT to detect ischemia were not significantly different across CAC score categories.^90^ While FFR-CT performance declined as the CAC score increased, it had superior diagnostic performance compared to CCTA across CAC score ranges. In CREDENCE, the authors demonstrated excellent accuracy for AI-CCTA to detect high-grade stenosis with similar efficacy to lab-interpreted quantitative coronary angiography.^37,91^

Current evidence suggests FFR-CT performs well, given its noninvasive nature, ease of use, and reliability, without the need for additional imaging sequences or pharmacological stress agents. It has strong diagnostic accuracy when compared to invasive FFR, and its emphasis on predicting flow obstruction makes it a vital tool for clinical decision-making in CAD. Limitations of FFR-CT include limited data for assessment of nonnative coronary arteries including stenting and bypass.

## Limitations of AI-CCTA

Limitations of AI stem from the fact that AI models are still in early investigation and need ongoing optimization to ensure reliable performance across diverse clinical environments and patient populations. This is large due to the nature of how AI-CCTA algorithms are built, relying on pre-existing ML databases to extrapolate and interpret new images.^18^ AI-based systems tend to be less reliable when presented with rare or complex imaging findings that were not adequately represented in the corresponding training data. This can lead to misinterpretations or missed diagnoses, reinforcing the need for human oversight when utilizing AI. Relying on a pre-existing database also opens the risk to create biases in data interpretation, by virtue of using training data not representative of the general population when generating models. Finally, while many AI-CCTA studies have demonstrated promising results in controlled environments, more large-scale real-world validation studies are needed to fully capture the variability of actual clinical settings, such as with the CONFIRM-2 database.

Such limitations have led to concerns regarding the capacity to rely on machine learning databases for CCTA interpretation across heterogeneous clinical settings, due to possible drops in performance when facing increased variability. The CLARIFY trial investigated this concern by comparing interpretation variability using an AI-driven CCTA algorithm with expert human readers. While interpretations between AI and human experts were consistent within their groups, the trial did demonstrate higher variability across human readers when compared to AI-CCTA.^34^ To this point, the SMART-CT trial investigated the potential of AI to improve CCTA interpretation in busy clinical settings. SMART-CT demonstrated reduced CCTA interpretation time and inter-reader variability across expert readers using AI assistance.^92^ These trials reinforce how AI can improve efficacy by enhancing clinical performance and reducing variability without replacing human expert readers.

## Conclusion

With the incorporation of CCTA in several major national and international guidelines for cardiovascular disease and marked improvement in the quality of image acquisition, the utilization of CCTA in modern medicine is expected to increase significantly. While conventional data processing techniques have improved efficiency and radiation dose in the last decade, more recent studies investigating AI in CCTA data processing have shown even greater achievements in speed, consistency, and reduction of radiation exposure. CCTA is an imaging modality that captures a high bandwidth of data, and AI-assisted data processing demonstrates great promise in comprehensive and objective image analysis allowing for higher quality and individualized decision-making, prognostication, risk analysis, and cardiovascular outcome prediction. AI presents the opportunity for a more complete analysis of CCTA data to reduce major cardiovascular events by better clarifying factors driving different plaque sub-types and their progression to worsening CAD. As AI will always need qualified human confirmation and validation, utilization of AI does not seek to replace physician contribution, but rather to enhance it via improvements in imaging innovation that drive preventative, periprocedural, and interventional stages of cardiovascular care. AI remains limited due to its early phase in the investigation, the need for further improvements in AI modeling, possible medicolegal implications, and the need for further large-scale validation studies such as the ongoing CONFIRM-2 trial.^93^ Despite these limitations, AI-CCTA represents a vital opportunity for improving cardiovascular care in an increasingly advanced and data-driven world of modern medicine.

## Declarations

**Ethics approval and consent to participate:** Not applicable.

**Consent for publication:** Not applicable.

**Availability of data and materials:** Not applicable.
